# Small molecule inhibition of group I p21-activated kinases in breast cancer induces apoptosis and potentiates the activity of microtubule stabilizing agents

**DOI:** 10.1186/s13058-015-0564-5

**Published:** 2015-04-23

**Authors:** Christy C Ong, Sarah Gierke, Cameron Pitt, Meredith Sagolla, Christine K Cheng, Wei Zhou, Adrian M Jubb, Laura Strickland, Maike Schmidt, Sergio G Duron, David A Campbell, Wei Zheng, Seameen Dehdashti, Min Shen, Nora Yang, Mark L Behnke, Wenwei Huang, John C McKew, Jonathan Chernoff, William F Forrest, Peter M Haverty, Suet-Feung Chin, Emad A Rakha, Andrew R Green, Ian O Ellis, Carlos Caldas, Thomas O’Brien, Lori S Friedman, Hartmut Koeppen, Joachim Rudolph, Klaus P Hoeflich

**Affiliations:** Department of Translational Oncology, Genentech, Inc., South San Francisco, CA USA; Department of Pathology, Genentech, Inc., South San Francisco, CA USA; Department of Diagnostics, Genentech, Inc., South San Francisco, CA USA; Afraxis, La Jolla, CA USA; National Center for Advancing Translational Sciences, Bethesda, MD USA; Fox Chase Cancer Center, Philadelphia, PA USA; Department of Biostatistics, Genentech, Inc., South San Francisco, CA USA; Department of Bioinformatics, Genentech, Inc., South San Francisco, CA USA; Cancer Research UK, University of Cambridge, Cambridge, UK; Histopathology, Division of Cancer and Stem Cells, School of Medicine, University of Nottingham and Nottingham University Hospitals, Nottingham, UK; Discovery Chemistry, Genentech, Inc., South San Francisco, CA USA; New address: University of California, San Francisco, CA USA; New address: COI Pharmaceuticals, La Jolla, CA USA; New address: Food and Drug Administration, Silver Spring, MD USA; New address: aTyr Pharma, San Diego, CA USA; New address: Blueprint Medicines, Cambridge, MA UK

## Abstract

**Introduction:**

Breast cancer, the most common cause of cancer-related deaths worldwide among women, is a molecularly and clinically heterogeneous disease. Extensive genetic and epigenetic profiling of breast tumors has recently revealed novel putative driver genes, including p21-activated kinase (PAK)1. PAK1 is a serine/threonine kinase downstream of small GTP-binding proteins, Rac1 and Cdc42, and is an integral component of growth factor signaling networks and cellular functions fundamental to tumorigenesis.

**Methods:**

PAK1 dysregulation (copy number gain, mRNA and protein expression) was evaluated in two cohorts of breast cancer tissues (n = 980 and 1,108). A novel small molecule inhibitor, FRAX1036, and RNA interference were used to examine PAK1 loss of function and combination with docetaxel *in vitro*. Mechanism of action for the therapeutic combination, both cellular and molecular, was assessed via time-lapse microscopy and immunoblotting.

**Results:**

We demonstrate that focal genomic amplification and overexpression of PAK1 are associated with poor clinical outcome in the luminal subtype of breast cancer (*P* = 1.29 × 10^−4^ and *P* = 0.015, respectively). Given the role for PAK1 in regulating cytoskeletal organization, we hypothesized that combination of PAK1 inhibition with taxane treatment could be combined to further interfere with microtubule dynamics and cell survival. Consistent with this, administration of docetaxel with either a novel small molecule inhibitor of group I PAKs, FRAX1036, or PAK1 small interfering RNA oligonucleotides dramatically altered signaling to cytoskeletal-associated proteins, such as stathmin, and induced microtubule disorganization and cellular apoptosis. Live-cell imaging revealed that the duration of mitotic arrest mediated by docetaxel was significantly reduced in the presence of FRAX1036, and this was associated with increased kinetics of apoptosis.

**Conclusions:**

Taken together, these findings further support PAK1 as a potential target in breast cancer and suggest combination with taxanes as a viable strategy to increase anti-tumor efficacy.

**Electronic supplementary material:**

The online version of this article (doi:10.1186/s13058-015-0564-5) contains supplementary material, which is available to authorized users.

## Introduction

The p21-activated kinases (PAKs) have generated significant interest as therapeutic targets in cancer [1,2]. The PAK family is comprised of six members and is subdivided into two groups (Groups I and II) based on sequence and structural homology. PAKs are currently amongst the most well-characterized effector proteins of the Ras-related C3 botulinum toxin substrate 1 (Rac) and cell division control protein 42 (Cdc42). These GTPases stimulate PAK catalytic activity by relieving an intramolecular interaction between the kinase and autoinhibitory domains. The kinase domains of Group I versus II PAKs share approximately 50% identity and also share homology with additional members of the sterile-20 (STE20) subfamily of the kinome that are upstream activators of mammalian mitogen-activated protein kinase (MAPK) pathways.

PAK1 signaling has been shown to be important for regulating cytoskeletal organization and cell migration via both its catalytic activity and protein-protein interactions. For instance, PAK1 modulates the activity of myosin II (an actin interacting motor protein that can drive cell contractility), LIM-kinase (involved in actin polymerization through inactivation of cofilin family proteins) and filamin A (a large actin-binding protein that induces membrane ruffling) [3]. PAK1 is also involved in the phosphorylation of proteins that control microtubule dynamics such as stathmin, which destabilizes microtubules by binding tubulin dimers to inhibit tubulin polymerization and promote microtubule disassembly [4]. In addition, PAK1 phosphorylates tubulin cofactor B to augment heterodimerization of tubulin [5] as well as dynein light chain 1 which is a component of the cytoplasmic dynein complex that moves along with microtubules [6]. To date, the evidence for the role of PAK1 in microtubule remodeling comes primarily from overexpression and genetic studies. For instance, PAK1^−/−^ mouse embryonic fibroblasts display decreased microtubule regrowth and polymerization compared with wild-type cells, and the reciprocal phenotypic was observed using MCF7 breast cancer cells overexpressing PAK1 [7]. The contribution of PAK1 catalytic activity to microtubule dynamics has yet to be thoroughly explored.

In addition to its role in regulation of the cytoskeleton, PAK1 has been implicated in cellular processes that directly contribute to tumorigenesis, including growth factor pathways, cell proliferation, and pro-survival signaling [8]. PAK1 is also an effector of well-established oncogenes, such as the Ras small monomeric GTPase which is mutated in approximately 30% of human tumors. Given that Rac and Cdc42 lie downstream of Ras [9,10], several groups have evaluated the contribution of PAKs to Ras-driven cellular transformation and *in vivo* tumorigenesis [11,12]. For instance, PAK1 deletion in a mouse model of Ras-driven cutaneous squamous cell carcinoma led to markedly decreased tumorigenesis and progression, which was accompanied by attenuated signaling through MAPK and cytoskeletal pathways [11].

In terms of direct dysregulation in cancer, PAK1 is amplified, overexpressed or hyperactivated in several tumor subtypes [1,13]. Of note, focal genomic amplification of PAK1 at 11q14.1 has been reported for hormone receptor-positive breast carcinoma [14,15]. Analysis of breast cancer cell lines with PAK1 genomic copy number gain using RNA interference approaches revealed dependence on PAK1 expression for cell survival [14] and transformation [16]. Consistent with these findings, functional studies using transgenic mouse models have also demonstrated that overexpression of PAK1 in the mammary gland promotes the formation of preneoplastic lesions and breast tumors [17] and that PAK1 contributes to human endothelial growth factor receptor 2 (HER2)/Neu-driven tumorigenesis [18].

However, given this emerging body of work, a detailed assessment of PAK1 copy number alteration and validation experiments using small molecule inhibitors to evaluate PAK1 catalytic inhibition in breast cancer are still lacking. Moreover, the potential efficacy of PAK1 inhibition in combination with additional inhibitors of cytoskeletal organization has yet to be examined. Herein, we demonstrate that PAK1 gene amplification and protein overexpression are associated with poor clinical outcome in a large collection of luminal breast cancers. We also introduce a novel ATP-competitive small molecule inhibitor of group I PAKs, FRAX1036, and demonstrate sensitivity of PAK1-amplified breast cancer cells to this compound. Taken together, these results suggest that further investigation of PAK1 as a therapeutic target in breast cancer is warranted. Given that PAK1 regulates the cytoskeleton and microtubule inhibitors are used as standard-of-care chemotherapy in advanced breast cancer, we explored the molecular and cellular mechanisms for this therapeutic combination and showed increased anti-tumor efficacy in breast cancer cells.

## Materials and methods

### Materials, cell culture and viability assays

FRAX1036 was synthesized by Afraxis, Inc. (La Jolla, CA, USA) and docetaxel was purchased from Selleck Chemicals (Houston, TX, USA). Antibodies used for immunoblotting (p-MEK1-S298, p-CRAF-S338, Cleaved PARP, Cyclin D1, p-Stathmin-S16, p-β-catenin-S675, MCL-1, BCL-xL, p-Bad-S112 and PAK1) were purchased from Cell Signaling Technology (Danvers, MA, USA); anti-Actin was purchased from Sigma (St Louis, MO, USA). Cell lines were acquired from the American Type Culture Collection (ATCC; Manassas, VA, USA) and maintained at 37°C and 5% CO_2_ in RPMI 1640 media with 10% fetal bovine serum and 2 mM L-glutamine. U2OS-red fluorescent protein (RFP)-Tubulin cells (Marinpharm, Luckenwalde, Germany) were stably transduced with a plasmid expressing green fluorescent protein (GFP)-histone H2B. Cell transfections and treatments were performed using short interfering RNA oligonucleotides for PAK1 from Dharmacon RNAi Technologies (Chicago, IL, USA). Cellular viability was assessed via ATP content using the CellTiter-Glo Luminescent Assay (Promega, Madison, WI, USA) and results represent mean ± standard deviation from three experiments.

### PAK1/CCND1 survival analysis

Breast tumors from the Molecular Taxonomy of Breast Cancer International Consortium (METABRIC) dataset [15] with survival and DNA copy number data were selected, yielding 980 patients. DNA copy number was calculated using Affymetrix SNP6.0 arrays and a modified version of the PICNIC algorithm [19], published recently [20]. Samples were identified as having amplification of either *PAK1* or *CCND1* if the absolute copy number of the respective gene was >5 copies. The Kaplan-Meier plot and log-rank test were performed using the censored survival values (days since diagnosis) provided with the METABRIC dataset and our calculated *PAK1* amplification status using the R language [21], version 3.1, and the R package “survival”, version 2.37-7.

A Cox proportional hazard model was constructed using the METABRIC censored survival data, Nottingham prognostic index (NPI), patient age, and patient PAM50 breast cancer subtype classification in addition to the interaction of *CCND1* and *PAK1* amplification statuses. More specifically, the model “survival ~ NPI + age + PAM50 + *CCND1* * *PAK1*” was fit using the “coxph” R package, where ccnd1 and pak1 are binary variables, as discussed above. The forest plot was produced using the coefficients from this model and their *P*-values. The whiskers on this plot represent ±1.96 × the standard error for each coefficient. The coefficient for amplification of both *CCND1* and *PAK1* (dual amplification) in the same sample was calculated as the sum of the coefficients “pak1Amplified”, “ccnd1Amplified”, and the coefficient for the interaction term for these two terms.

### Bliss analysis

Cellular viability was assessed via ATP content using the CellTiter-Glo Luminescent Assay (Promega, Fitchburg, WI, USA) after a 4-day incubation period, and results represent mean ± standard deviation from three experiments. Total luminescence was measured on a Wallac Multilabel Reader (Perkin-Elmer, Waltham, MA, USA). Cells were treated simultaneously with FRAX1036 (dose range = 0 to 5 μM) or docetaxel (dose range = 0 to 0.4 nM) in an 8 × 10 matrix of concentrations. Combination synergy of FRAX1036 and docetaxel was determined by Bliss independence analyses. A Bliss expectation for a combined response C was calculated by the equation: C = (A + B) - (A × B) where A and B are the fractional growth inhibitions of given doses of drug A and B. ΔBliss scores were summed across the dose matrix to generate a Bliss sum. Bliss sum = 0 indicates that the combination effect is additive while Bliss sum >0 indicates synergy effect and Bliss sum <0 indicates antagonism effect. Statistical analysis comparing the Bliss sums for each cell line was conducted by the Student’s *t* test.

### Biochemical assays

The activity/inhibition of human recombinant PAK1 (kinase domain), PAK2 (full length) or PAK4 (kinase domain) was estimated by measuring the phosphorylation of a FRET peptide substrate (Ser/Thr19) labeled with Coumarin and Fluorescein using Z’-LYTE™ assay (Invitrogen, Carlsbad, CA, USA). The 10 μL assay mixtures contained 50 mM HEPES (pH 7.5), 0.01% Brij-35, 10 mM MgCl_2_, 1 mM EGTA, 2 μM FRET peptide substrate, and PAK enzyme (20 pM PAK1; 50 pM PAK2; 90 pM PAK4). Incubations were carried out at 22°C in black polypropylene 384-well plates (Corning Costar, Corning, NY, USA). Prior to the assay, enzyme, FRET peptide substrate and serially diluted test compounds were preincubated together in assay buffer (7.5 μL) for 10 minutes, and the assay was initiated by the addition of 2.5 μL assay buffer containing 4× ATP (160 μM PAK1; 480 μM PAK2; 16 μM PAK4). Following the 60-minute incubation, the assay mixtures were quenched by the addition of 5 μL of Z’-LYTE™ development reagent, and 1 hour later the emissions of Coumarin (445 nm) and Fluorescein (520 nm) were determined after excitation at 400 nm using an Envision plate reader (Perkin Elmer). An emission ratio (445 nm/520 nm) was determined to quantify the degree of substrate phosphorylation.

### Immunoblotting

Protein extracts were prepared at 4°C with RIPA Lysis Buffer (EMD Millipore Corporation, Billerica, MA, USA), 1 mM phenylmethylsulphonyl fluoride, Phosphatase Inhibitor Cocktail 2/3 and protease inhibitor cocktail (Sigma-Aldrich). For Western blot analysis, proteins were resolved by 4 to 12% SDS-PAGE and transferred to nitrocellulose membranes (Life Technologies, Grand Island, NY, USA). Immunoblotting was performed using the indicated primary antibodies and analyzed using secondary antibodies for enhanced chemiluminescence.

### IncuCyte apoptosis assays

For caspase 3/7 activation apoptosis assays, cells were plated at 10,000 cells/well in 96-well Corning plates for 24 hours prior to treating with DMSO, FRAX1036, and/or docetaxel. Caspase 3/7 reagent was added at a 1:1000 dilution (Essen Bioscience No. 4440, Ann Arbor, MI, USA). Cells were imaged at 10× magnification in an IncuCyte Zoom Live-content imaging system (Essen Bioscience) at 37°C, 5% CO_2_. Images were acquired every 2 hours or 4 hours for 36 to 72 hours, two images/well. Data was analyzed using IncuCyte analysis software to detect and quantify green (apoptotic) cells/image. Each condition was performed in triplicate. Averages with SEM at each time point were plotted in Excel (Microsoft, Redmond, WA, USA). A *t*-test was performed for the final time point comparing the combination of FRAX1036 and docetaxel with each single agent in Prism (Graphpad, La Jolla, CA, USA). The apoptotic index was calculated from the apoptosis assays by dividing the final apoptotic cell count by the total cell count. Averages with SEM were plotted in Excel (Microsoft), and a *t*-test was performed comparing the combination of FRAX1036 and docetaxel with each single agent in Prism (Graphpad).

### Live-cell microscopy and image analysis

U2OS cells stably expressing GFP-Histone H2B and RFP-Tubulin were cultured in Dulbecco’s modified Eagle’s high glucose medium, 10% fetal bovine serum, NEAA, at 37°C and 5% CO_2_. For live imaging experiments, U2OS cells were plated in a 24-well glass bottom, black-walled plate (Sensoplate #662892, Greiner Bio-one, Monroe, NC, USA). On the following day, cells were treated with DMSO, FRAX1036, and/or docetaxel and imaged every 10 minutes for 72 hours with a 40× ELWD Plan Fluor objective (NA: 0.6, Nikon, Tokyo, JP) at 37°C and 5% CO_2_. Imaging was performed on a Nikon Ti-E perfect focus inverted microscope equipped with a spinning disk confocal CSU-X1 (Andor, Oxford Instruments, Abingdon, Oxfordshire, UK), motorized X,Y stage (Nikon), environmental chamber (OkoLab, Burlingame, CA, USA) and iXon3 897 EMCCD camera or Clara interline CCD camera (Andor, Oxford Instruments), all controlled by NIS-Elements software (Nikon, Tokyo, JP). Time-lapses were analyzed in NIS-Elements, and supplemental movies were generated in Quicktime Pro (Apple, Cupertino, CA, USA). For high-resolution imaging of microtubule organization, U2OS cells were imaged after 20 hours treatment with a 60× Plan Apo objective (NA: 1.4, Nikon). For immunofluorescence, MDA-MB-175 cells were methanol fixed, permeabilized in TBS-0.5% TritonX-100 and blocked in 2% bovine serum albumin, 0.1% Triton X-100. Microtubules were probed with primary antibody rat-anti tubulin (1:250, Serotec clone YL1/2), secondary antibody Alexafluor488 anti-rat (1:500, A-11006, Life Technologies, Grand Island, NY, USA), and mounted with Prolong Gold with DAPI (Life Technologies, Grand Island, NY, USA).

For the duration of mitosis/mitotic arrest and cell fate measurements, cells were monitored from the time they began to round up from the plate to the time when they were observed to divide, slip out of mitosis with micronuclei, or apoptose. Observations were made using the phase morphology of the cells as well as chromosome condensation/decondensation and mitotic spindle morphology in the fluorescent channels. Cells that divided or slipped were monitored for the remainder of the 72-hour movie, and subsequent cell events were recorded. Duration of mitosis/mitotic arrest was graphed in Prism (GraphPad), and significance was determined by one-way analysis of variance with multiple comparisons to compare each condition to one another. A *t*-test was performed on the two significant but close conditions (docetaxel and FRAX1036 + docetaxel).

## Results

### *PAK1* amplification and overexpression are associated with poor outcome in luminal breast cancer

**Figure 1:**
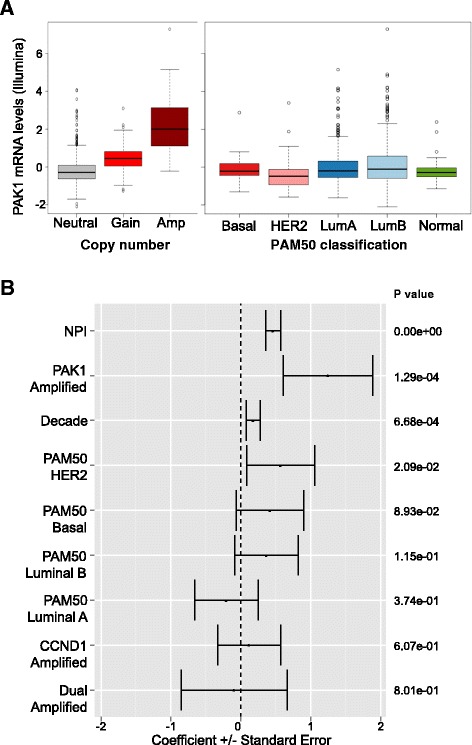
p21-Activated kinase (PAK)1 copy number and expression is elevated and associated with poor clinical outcome in breast tumors analyzed by the Molecular Taxonomy of Breast Cancer International Consortium (METABRIC). **(A)** Illumina mRNA expression of PAK1 is correlated with copy number alteration and breast cancer subtype in METABRIC tissue samples. “Amplification” is defined as gene amplification greater than or equal to 5 copies, while “Gain” is defined as >2 and <5 copies. **(B)** PAK1 focal amplification is associated with poor patient prognosis. Hazard ratio coefficient (log_2_ scale) is plotted for clinical and molecular parameters. Hazard ratio is significantly higher for PAK1 focal amplification relative to CCND1 focal amplification or dual PAK1/CCND1 amplification in luminal breast tumors (n = 980). *P* values are shown. CCND1, cyclin D1; Decade, patient age at time of diagnosis; Lum A, luminal A; Lum B, luminal B; NPI, Nottingham prognostic index.

The genomic and transcriptomic architecture of 2,000 breast tumors was recently characterized as part of METABRIC [15]. Clustering analysis of joint copy number and gene expression data from the *cis-*associated genes revealed 10 novel molecular subgroups for breast cancer, including an estrogen receptor-positive subgroup composed of amplification at 11q13/14. This amplified region includes *CCND1* (11q13.3) and *PAK1* (11q14.1). Consistent with previous reports [14], PAK1 mRNA expression was correlated with copy number gain (Figure 1A) and elevated in luminal breast cancer subtypes (Figure 1B) in METABRIC samples. To determine the prognostic significance of PAK1 in breast cancer, gene amplification was correlated with clinical outcome using a Cox proportional hazard model constructed with METABRIC censored survival data, patient age, NPI [22], PAM50 breast cancer subtype classification, and *CCND1*/*PAK1* amplification status. As expected, the NPI score, patient age and certain PAM50 subtypes were associated with worse hazard ratio (Figure 1B). High level, focal *PAK1* amplification was significantly associated with poor patient outcome (*P* = 1.29 × 10^−4^) (Figure 1B; Additional file 1: Figure S1A), although this was not noted for either focal gain of *CCND1* or broad amplification of both *CCND1* and *PAK1*. Furthermore, elevated PAK1 mRNA expression was also correlated with poor survival in the same tissue samples (data not shown).

The association of PAK1 dysregulation with survival of breast cancer patients was evaluated at the protein level in an independent sample set of 1,108 estrogen receptor-positive, early-stage breast tumors. PAK1 immunohistochemistry was validated previously [14] and breast tumor tissues were analyzed on a standard histology score of 0 to 3. Patients whose tumors had the lowest protein expression of PAK1 (staining intensity 0 to 1) displayed better overall survival than patients whose tumors had high PAK1 expression levels (staining intensity 2 to 3) (Additional file 1: Figure S1B; two group hazard ratio = 0.80). Taken together, these results indicate that PAK1 genomic amplification and overexpression are correlated with poor patient outcome in luminal breast cancer.

### FRAX1036 combines with docetaxel to alter signaling to microtubule regulators and induce apoptotic markers in luminal breast cancer lines

**Figure 2:**
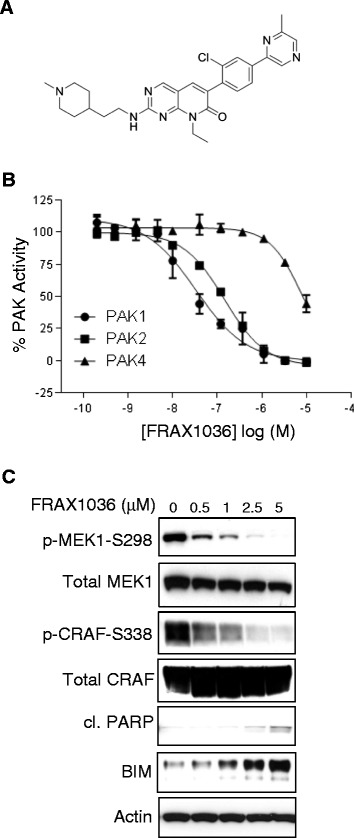
FRAX1036 inhibition of group I p21-activated kinase (PAK) isoforms. **(A)** Chemical structure of the group I PAK inhibitor, FRAX1036. **(B)** Concentration-response analysis of FRAX1036 against PAK1, PAK2 or PAK4. Concentration response curves were generated in duplicate and represent one of at least three experiments for PAK1 and PAK2 with similar results. Data shown for PAK4 represent one of two experiments with similar results. Each curve is normalized to zero and 100% based on no enzyme or DMSO, respectively. **(C)** Pharmacodynamic changes induced by FRAX1036 dose–response. MDA-MB175 cells were treated with increasing concentrations of FRAX1036 for 24 hours. Cell lysates were immunoblotted with antibodies against biomarkers involved in PAK1 effector and survival signaling.

The small molecule pyridopyrimidinone inhibitor FRAX1036 (Figure 2A) was derived from chemical optimization of arylamino pyridopyrimidinone PAK inhibitors, as represented by FRAX597 [2,23]. FRAX1036 is devoid of the characteristic arylamino moiety of earlier generation compounds, resulting in improved kinase selectivity (Additional file 2: Table S1) and general drug properties. Its biochemical potency (Ki) against PAK1 and PAK2 is 23.3 and 72.4 nM, respectively, with high selectivity against PAK4 (Ki = 2.4 μM; Figure 2B). In order to test the cellular activity of FRAX1036, breast cancer cell lines with known *PAK1* gene amplification status were tested for levels of PAK1 expression and activity (Additional file 3: Figure S2). Potent cellular inhibition of group I PAK substrate phosphorylation (MEK1-S298 and CRAF-S338) was observed at 2.5 to 5 μM concentrations of FRAX1036 in *PAK1*-amplified MDA-MB-175 cells (Figure 2C). Consistent with previous reports evaluating PAK1 function in breast cancer cell lines via genetic approaches [9], dose-dependent inhibition of PAK1 effector signaling was correlated with poly(ADP-ribose) polymerase (PARP) cleavage.

**Figure 3:**
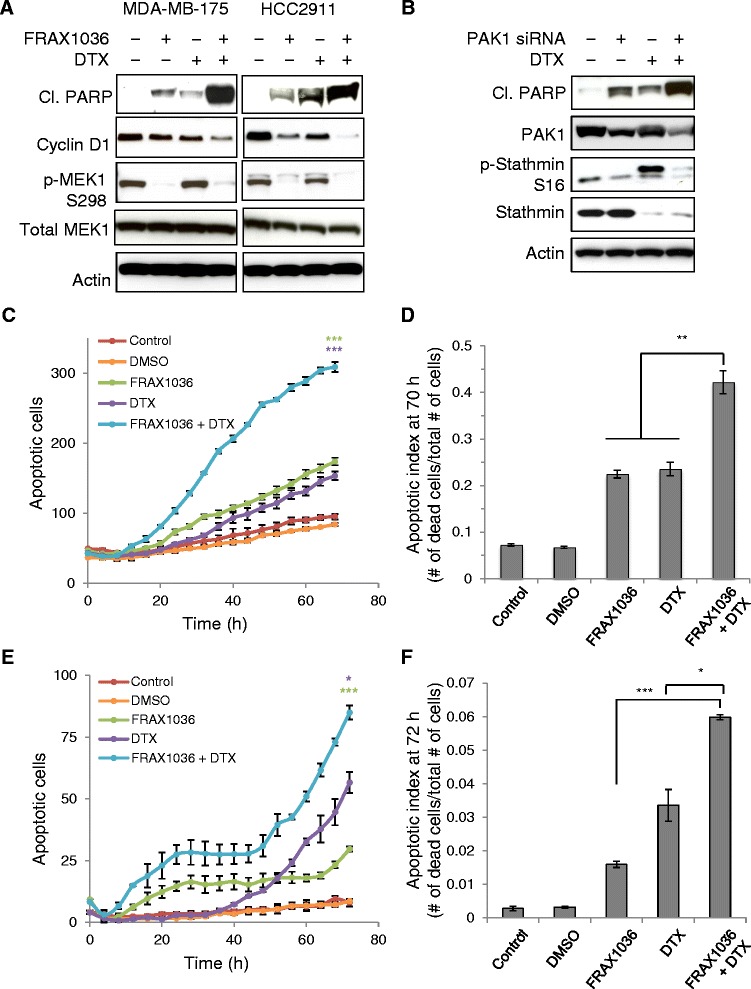
FRAX1036 and docetaxel (DTX) combine to alter stathmin phosphorylation, induce the apoptotic marker cleaved PARP and increase kinetics of apoptosis. **(A)** MDA-MB-175 and HCC2911 cells were treated with DMSO, 5 μM FRAX1036, 0.2 μM docetaxel and a combination of 5 μM FRAX1036 and 0.2 μM docetaxel for 24 hours. Cell lysates were immunoblotted with apoptotic and PAK1 downstream markers. **(B)** MDA-MB-175 cells were treated with DMSO or 0.2 μM docetaxel for 48 hours after non-targeting control short interfering RNA (siRNA) or PAK1 siRNA transfection for 72 hours. Cell lysates were harvested and subjected to immunoblot analysis for apoptotic markers and microtubule regulators. The molecular weight of the lower band from the phospho-stathmin immunoblot corresponds to total stathmin. The efficacy of knockdown by PAK1 siRNA was 47% (lane 2) and 80% (lane 4) as determined by densitometry. **(C)** Kinetic apoptosis assay. HCC2911 cells were plated in 96-well plates and were untreated (control) or treated with DMSO, 2.5 μM FRAX1036, 0.2 μM docetaxel, or a combination of 2.5 μM FRAX1036 and 0.2 μM docetaxel. Apoptosis was assayed by counting the number of green caspase 3/7-positive objects at each time point (Essen Cell player kinetic caspase 3/7 assay). **(D)** Apoptotic index. The number of apoptotic cells was normalized to the total number of cells at the final time point in (C) to account for cell proliferation. **(E**,**F)** The same as (C,D) with MDA-MB-175 cells. The average and SEM of three replicates are shown and a *t*-test performed at the final time point and on the apoptotic index (*P < 0.03, **P < 0.003, ***P ≤ 0.0001).

The PAK effector, stathmin, is a microtubule destabilizing protein and phosphorylation at serine 16 by PAK and other kinases, and regulates stathmin-tubulin binding [18,24]. We therefore hypothesized that PAK1 inhibition in combination with microtubule stabilizing chemotherapeutic agent taxanes, such as docetaxel (Taxotere, DTX), could synergistically alter microtubule dynamics in breast cancer cells leading to greater cell death [25]. FRAX1036 and docetaxel combination treatment of PAK1-amplified lines, MDA-MB-175 and HCC2911, elevated a major apoptotic marker (cleaved PARP) and attenuated a cell cycle regulator (cyclin D1) (Figure 3A). Single-agent docetaxel treatment increased stathmin-S16 phosphorylation indicative of the accumulation of cells in mitosis [26]. To further validate the observation of a combined effect of PAK1 inhibition and microtubule perturbation, the combination of docetaxel with PAK1 short interfering RNA knockdown was also investigated (Figure 3B). PAK1-dependent phosphorylation of stathmin was observed in breast cancer cells following selective knockdown (Figure 3B) or FRAX1036 treatment (Additional file 4: Figure S2B). Comparable apoptotic signaling changes were observed for either PAK1 small molecule inhibition or knockdown in combination with docetaxel. Since combined inhibition altered signaling to caspase substrates (cleaved PARP), we further examined cell viability by monitoring apoptosis using high content time-lapse imaging. HCC2911 and MDA-MB-175 breast cancer cells treated with the combination of FRAX1036 and docetaxel showed increased kinetics of apoptosis compared with either single agent (Figure 3C,E). After 72 hours of treatment, the apoptotic index (ratio of apoptotic cells/total cells) was analyzed to account for cell proliferation (Figure 3D,F). FRAX1036 and docetaxel combination treatment resulted in a significantly greater apoptotic index over FRAX1036 or docetaxel alone in both cell lines. Synergistic modulation of cell viability was also demonstrated via FRAX1036 and docetaxel dose ranging and Bliss independence analysis (Additional file 4: Figure S3). Taken together, these data indicate that combinatorial increases in tumor cell killing are observed with FRAX1036 and docetaxel treatment.

### FRAX1036 and docetaxel combination alter microtubule organization, duration of mitotic arrest and kinetics of apoptosis

**Figure 4:**
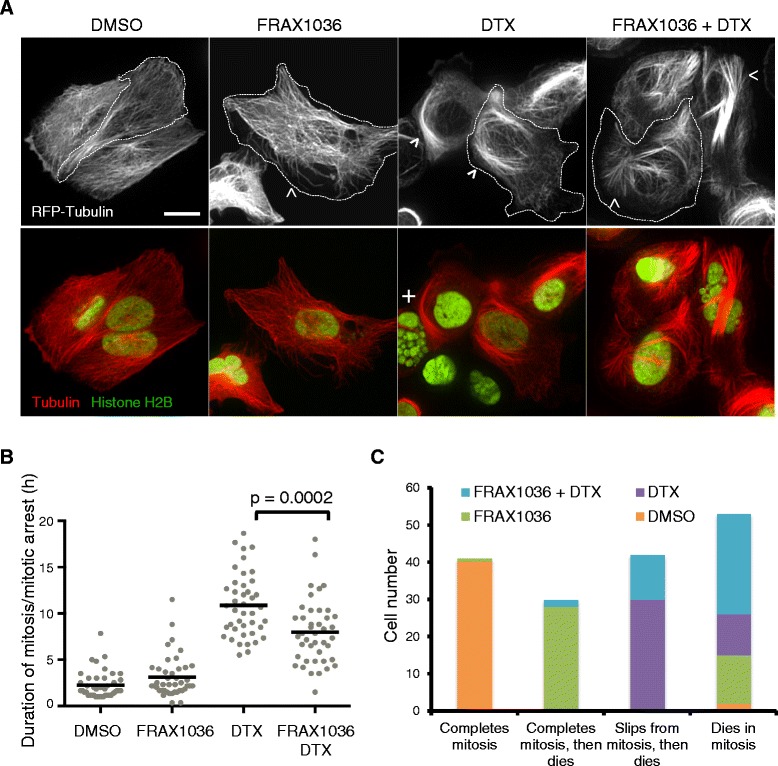
FRAX1036, docetaxel (DTX) and their combination affects microtubule organization, mitosis and cell fate. **(A)** Spinning-disk confocal images of live U2OS cells expressing red fluorescent protein (RFP)-Tubulin (red) and green fluorescent protein-Histone H2B (green) bottom panel. The top panel is RFP-Tubulin channel alone with an individual cell outline by a dotted line for each condition. Arrows highlight changes in microtubule organization that are characteristic of each treatment. A micronucleated cell is indicated by +. Cells were treated with DMSO, 2.5 μM FRAX1036, 0.2 μM docetaxel, or a combination of 2.5 μM FRAX1036 and 0.2 μM docetaxel for 20 hours before imaging. Scale bar = 20 μm. **(B)** Duration of mitosis/mitotic arrest of cells treated with DMSO, 2.5 μM FRAX1036, 0.2 μM docetaxel, or a combination of 2.5 μM FRAX1036 and 0.2 μM docetaxel. Cells were followed from time of entering mitosis to the time of division, slippage or apoptosis. Each grey symbol represents a single cell and black bars represent the average. N = 42 mitotic cells imaged from five fields of view. Data is from one of two experiments with similar results. One-way analysis of variance with multiple comparisons showed that all averages are significantly different except for DMSO:FRAX1036. A *t*-test was performed on FRAX1036 + DTX combination vs DTX alone (*P* = 0.0002). **(C)** Distribution of major cell fates after entry into mitosis of U2OS cells treated with FRAX1036, docetaxel and their combination. N = 42 mitotic cells for each treatment condition.

Both PAK1 signaling and docetaxel were previously reported to affect microtubule dynamics and progression through mitosis [25,27]. Regulation of microtubule dynamics ultimately affects microtubule length and organization of microtubule arrays. To directly visualize the effects of FRAX1036 and docetaxel treatments on microtubules, we utilized a U2OS osteosarcoma cell line that stably expresses RFP-Tubulin and GFP-Histone H2B. The flat and spread morphology of U2OS cells was more amenable to high-resolution microscopy, allowing us to visualize microtubules in live cells without fixation. Analysis of pharmacodynamic markers and apoptosis (Additional file 5: Figure S5A,B) confirmed that U2OS cells are similarly affected by FRAX1036, docetaxel and combination treatment as the breast cancer lines examined in this study. In DMSO-treated cells, microtubule arrays were organized by the microtubule organizing center and radiate uniformly to the cell periphery. After 20 hours of treatment with FRAX1036, microtubules were disorganized and were not evenly distributed throughout the cytoplasm, between the microtubule organizing center and the periphery (Figure 4A, arrow). As expected, docetaxel stabilized microtubules resulting in elongated bundles of microtubules that curved around the cytoplasm. Cells treated with both FRAX1036 and docetaxel had shorter, straight microtubules, suggesting a change in the regulation of microtubule dynamics from docetaxel treatment alone. In addition, immunofluorescence of fixed MDA-MB-175 cells treated with FRAX1036 and docetaxel showed similar effects on microtubule organization (Additional file 6: Figure S4). To further probe the relationship between cell cycle progression and apoptosis we imaged RFP-Tubulin and GFP-Histone H2B U2OS cells over a 72-hour treatment period using spinning disk confocal microscopy (Additional file 7: Movie 1). We tracked individual cells for each of the treatment conditions and visualized mitotic spindle formation to analyze timing and fate (division, slippage or cell death) after entering mitosis (Figure 4B,C; Additional file 5: Figure S5C). FRAX1036-treated cells completed normal mitoses with the majority of apoptosis occurring during interphase (66.7%). Because the cells are not synchronized it was not clear from our analysis whether a completed mitosis was required for apoptosis. Docetaxel-treated cells arrested in mitosis five-fold longer than control cells slipped out of mitosis (71.4%) without completing cell division and formed micronucleated cells that later died (Figure 4A; Additional file 5: Figure S5C, + symbol). In contrast, when FRAX1036 was combined with docetaxel, there was a small decrease in the duration of mitosis and the majority of these cells died during mitotic arrest (65.9%), possibly accounting for the increased rate of cell death. To confirm these results in breast cancer cells, we imaged MDA-MB-175 cells by phase-contrast microscopy (Additional file 8: Movie 2). Without clear visible markers of entry into mitosis, MDA-MB-175 cells were difficult to track and quantitate due to the densely packed and rounded morphology. However, the overall trends in cell fate could be observed: docetaxel treatment resulted in slipped cells with micronuclei that later died, while dead cells accumulated more quickly in the FRAX1036 and docetaxel combination. The dependency of FRAX1036 and docetaxel combination effects on the order of drug treatment was also determined. A pronounced decrease in cell viability was observed by simultaneous treatment of compounds and when docetaxel was dosed prior (4 hours) to FRAX1036 (Additional file 9: Figure S6).

## Discussion

The advent of high-throughput techniques for genetic and epigenetic characterization of tumor specimens has led to an exponential increase in our understanding of molecular events underlying the process of carcinogenesis. This is especially true for breast cancer, an indication in which tumor tissues can be successfully obtained and analyzed with high frequency. Typically, novel putative driver genes for breast cancer have been preliminarily evaluated using genetic and knockdown approaches. However, more comprehensive and rigorous assessment of intracellular targets for therapeutic intervention requires selective, potent and cell-active small molecules with good biochemical and cellular properties. FRAX1036 displays selectivity for PAK1-3 relative to group II PAK members as well as other kinases (Additional file 2: Table S1) and can be used as a tool compound for *in vitro* target validation experiments.

Interestingly, PAK1 genomic amplification or protein overexpression are strongly associated with poor outcome for luminal (or estrogen receptor-positive) breast cancer patients (Figure 1; Additional file 1: Figure S1). A subset of breast carcinomas without genomic amplification also display high mRNA and protein expression of PAK1 (Figure 1A; Additional file 1: Figure S1B). The molecular mechanisms underpinning dysregulated PAK1 expression in the absence of genomic amplification are not well characterized, although regulation by microRNAs [28] and gene translocation (Peter Haverty, unpublished data) have both been observed. *PAK1* copy number alterations have also been observed in other tumor indications, such as ovarian cancer and melanoma [16,29] and further validation efforts are necessary to apply the findings reported here to these other indications.

Hormone receptor-positive breast cancer patients with localized disease receive front-line treatment with endocrine therapies, such as tamoxifen or aromatase inhibitors. There is some evidence that PAK1 may directly phosphorylate estrogen receptor-α [30] or components of the estrogen receptor multi-protein complex [31]. However, the potential roles for PAK1 inhibition in combination with later lines of therapy, such as taxanes, have yet to be explored. Given the evolutionarily conserved role of PAK1 in regulating cytoskeletal dynamics and the common use of microtubule inhibitors in later lines of breast cancer treatment, we evaluated the mechanism and potential therapeutic benefit of FRAX1036 combination with docetaxel. We show that signaling changes elicited by FRAX1036 and docetaxel potentiate apoptosis of breast cancer cells and that microtubule morphology is affected by both pathways (Figure 4A). Combination treatment of FRAX1036 significantly diminished time in docetaxel-induced mitotic arrest (Figure 4C; Additional file 5: Figure S5), pushed cell fate from mitotic slippage to apoptosis (Figure 4B) and accordingly increased the kinetics of breast tumor cell apoptosis (Figure 3C-F; Additional file 3: Figure S2). Given that luminal breast cancer patients generally do not respond durably to chemotherapy, combination of FRAX1036 with taxanes may help address unmet needs for patients with advanced and metastatic disease.

## Conclusions

PAKs have been implicated in various aspects of tumorigenesis. In this study, we demonstrate that *PAK1* amplification and overexpression are associated with poor clinical outcome in a large collection of luminal breast cancers. Treatment of PAK1-amplified breast cancer cells with a novel small molecule inhibitor of group I PAKs, FRAX1036, resulted in apoptosis. Efficacy was also potentiated in combination with microtubule inhibitors that are used as standard-of-care chemotherapy in advanced breast cancer. Taken together, our findings support the further therapeutic evaluation of PAK inhibitors in breast cancer.

## Additional files

Additional file 1: Figure S1. PAK1 dysregulation is associated with poor patient outcome in breast cancer. **(A)** Kaplan-Meier curve for PAK1 amplification status in METABRIC breast tumor samples. Amplified is defined as *PAK1* gene amplification >5 copies. Survival differences between patients was statistically significant (*P* = 0.0156). **(B)** Association of PAK1 protein expression with clinical outcome. PAK1 immunohistochemistry (IHC) was performed as described previously (Ong and colleagues [29]) using breast cancer tissue microarrays (n = 1,108) provided by the University of Nottingham. Staining intensity was scored for replicate cores on a histology score of 0 to 3. Overall survival was plotted for patients with either low (score 0, 1) or high (score 2, 3) PAK1 tumor expression. Additional file 2: Table S1. Kinase inhibitory activities for FRAX1036 tested at 1 μM concentration. Biochemical assays were performed to determine the extent of FRAX1036 inhibition of 247 purified kinases. Data is shown for <50%, 50 to 75% and >75% inhibition at a compound concentration of 1 μM. Additional file 3: Figure S2. PAK1 levels and modulation of stathmin phosphorylation in breast cancer cells. **(A)** The protein expression level of PAK1, PAK2 and phospho-PAK1/2 were detected and compared in 15 breast cells. HCC2911 and MDA-MB-175 cells showed higher PAK1 activity and expression level. **(B)** FRAX1036 was administered to SUM52PE and T47D breast cancer cells at 0, 0.6, 1.2, 2.5, 5 and 10 μM for 24 hours. DMSO alone was used as a control. Additional file 4: Figure S3. Combination effects of FRAX1036 and docetaxel (DTX) on cell viability assay in HCC2911 and MDA-MB175 cells. **(A)** HCC2911 and **(B)** MDA-MB175 cells were treated with FRAX1036, DTX, or the combination of both drugs and measured in a 4-day Cell Titer-Glo assay. Percentage of inhibition for cell viability and ΔBliss data were calculated and plotted as a dose range for both drugs. Additional file 5: Figure S5 Kinetic apoptosis assay and live-cell microscopy were used to further determine the extent of apoptosis in response to FRAX1036, docetaxel (DTX), and a combination. **(A)** Kinetic apoptosis assay of U2OS cells treated as in Figure 3 and imaged every 2 hours. Average and SEM of three replicates are shown (**P* < 0.01). **(B)** U2OS cells were treated with 2 μM FRAX1036 and 0.2 μM DTX as indicated for 24 hours. Lysates were analyzed for proximal and distal pharmacodynamics biomarkers. **(C)** Time lapse images of U2OS cells stably expressing RFP-Tubulin and GFP-Histone H2B after treatment with DMSO, 2.5 μM FRAX1036, 0.2 μM docetaxel, or combination of 2.5 μM FRAX1036 and 0.2 μM DTX. Images are overlays of phase, RFP and GFP of a single field of view showing microtubules, nuclei and cell morphology from time-lapse movies (Additional file 7: Movie 1) at each time point. Symbols highlight different cell fates quantified in Figure 4C (*Mitotic; ^+^slipped with micronuclei; ^apoptotic). Scale bar = 20 μm. Additional file 6: Figure S4. Microtubule organization in MDA-MB-175 cells treated with FRAX1036 and DTX. Spinning-disk confocal immunofluorescence images of fixed MDA-MB-175 cells. Cells were treated with DMSO, 2.5 μM FRAX1036, 0.2 μM DTX, or a combination of 2.5 μM FRAX1036 and 0.2 μM DTX for 24 hours before fixation. Scale bar = 20 μm. Additional file 7: Movie 1. Live-cell microscopy of U2OS cells treated with FRAX1036, docetaxel (DTX), and a combination over 72 hours. Spinning disk confocal live-cell imaging of Phase, GFP-Histone H2B and RFP-Tubulin over 72 hours of treatment. Top-left panel: DMSO; top-right panel: 2.5 μM FRAX1036; bottom-left panel: 0.2 μM docetaxel; bottom-right panel: 2.5 μM FRAX1036 and 0.2 μM DTX. Images were acquired every 10 minutes for 72 hours. The video plays at 15 frames/seconds and is therefore accelerated 9,000 times. Additional file 8: Movie 2. Live-cell microscopy of MDA-MB-175 cells treated with FRAX1036, docetaxel (DTX) and a combination over 72 hours. Phase contrast live-cell imaging of: top-left panel, DMSO; top-right panel, 2.5 μM FRAX1036; bottom-left panel, 0.2 μM DTX; bottom-right panel: 2.5 μM FRAX1036 and 0.2 μM docetaxel. Images were acquired every 10 minutes for 72 hours using a 20× ELWD Plan Fluor objective (NA: 0.45, Nikon). The video plays at 15 frames/second and is therefore accelerated 9,000 times. Additional file 9: Figure S6. FRAX1036 and docetaxel order-of-addition modulates cell viability *in vitro*. Cell viability was quantified 48 hours following FRAX1036 and docetaxel (DTX) administered either simultaneously (bar 4), FRAX1036 preceding DTX by 4 hours (bar 5) or DTX preceding FRAX1036 by 4 hours (bar 6). The average and SEM of three replicates are shown. Simultaneous dosing and DTX followed by PAK1 inhibitor treatment were most efficacious (***P* < 0.001).

## Abbreviations

GFP: green fluorescent protein
MAPK: mitogen-activated protein kinase
METABRIC: Molecular Taxonomy of Breast Cancer International Consortium
NEAA: non-essential amino acids
NPI: Nottingham prognostic index
PAK: p21-activated kinase
PARP: poly(ADP-ribose) polymerase
RFP: red fluorescent protein
