# Supplementary material for: Small molecule inhibition of group I p21-activated kinases in breast cancer induces apoptosis and potentiates the activity of microtubule stabilizing agents
Source: Breast Cancer Res. 2015 Apr 23;17(1):59. doi: 10.1186/s13058-015-0564-5 (PMC4445529; doi:10.1186/s13058-015-0564-5)
Supplement: Additional file 1: Figure S1. — PAK1 dysregulation is associated with poor patient outcome in breast cancer. (A) Kaplan-Meier curve for PAK1 amplification status in METABRIC breast tumor samples. Amplified is defined as PAK1 gene amplification >5 copies. Survival differences between patients was statistically significant (P = 0.0156). (B) Association of PAK1 protein expression with clinical outcome. PAK1 immunohistochemistry (IHC) was performed as described previously (Ong and colleagues [29]) using breast cancer tissue microarrays (n = 1,108) provided by the University of Nottingham. Staining intensity was scored for replicate cores on a histology score of 0 to 3. Overall survival was plotted for patients with either low (score 0, 1) or high (score 2, 3) PAK1 tumor expression. [file 13058_2015_564_MOESM1_ESM.pptx]

## Slide 1
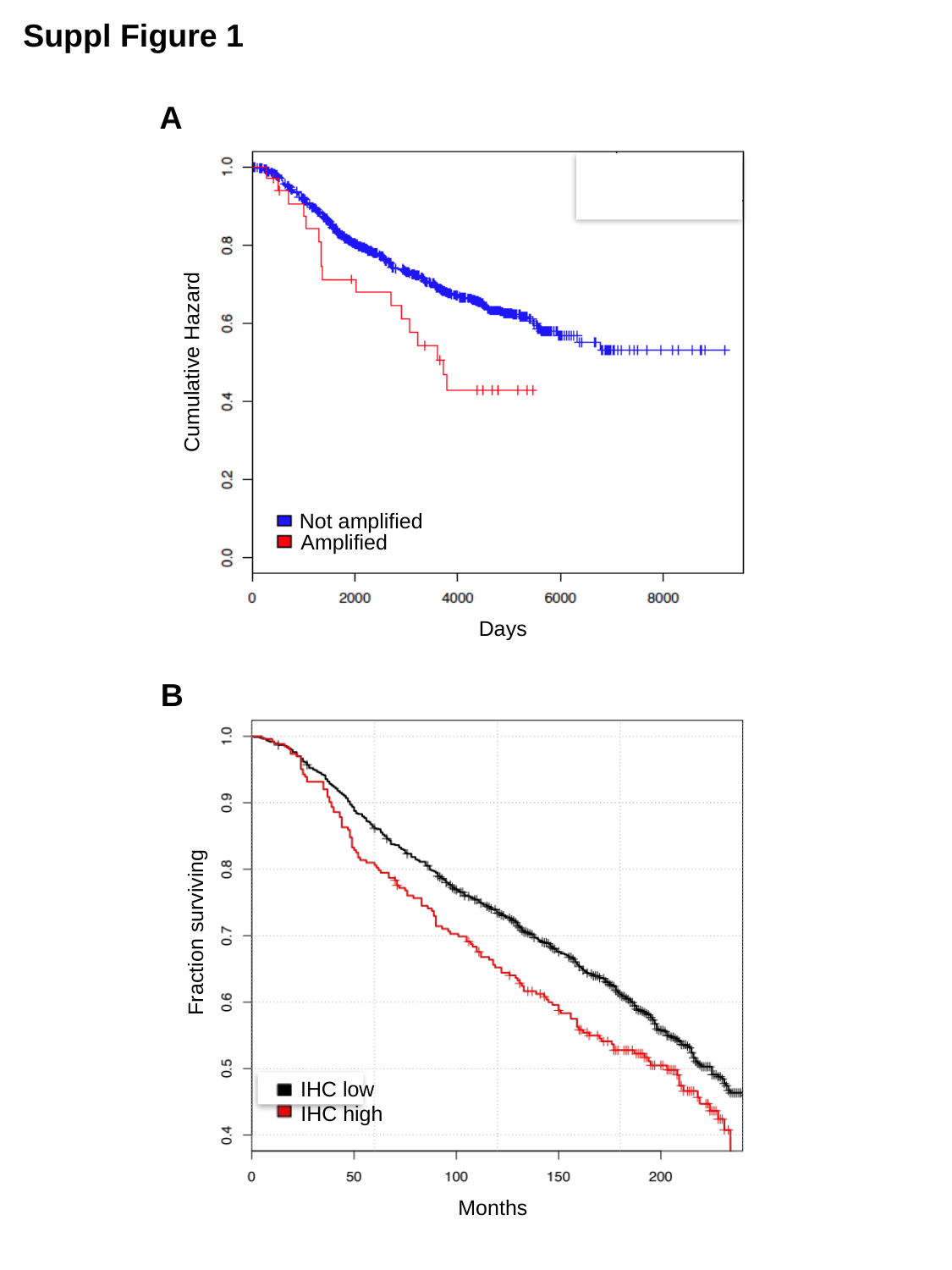

Suppl Figure 1
A
Cumulative Hazard
Not amplified
Amplified
Days
B
Fraction surviving
IHC low
IHC high
Months
