# Supplementary material for: Small molecule inhibition of group I p21-activated kinases in breast cancer induces apoptosis and potentiates the activity of microtubule stabilizing agents
Source: Breast Cancer Res. 2015 Apr 23;17(1):59. doi: 10.1186/s13058-015-0564-5 (PMC4445529; doi:10.1186/s13058-015-0564-5)
Supplement: Additional file 2: Table S1. — Kinase inhibitory activities for FRAX1036 tested at 1 μM concentration. Biochemical assays were performed to determine the extent of FRAX1036 inhibition of 247 purified kinases. Data is shown for <50%, 50 to 75% and >75% inhibition at a compound concentration of 1 μM. [file 13058_2015_564_MOESM2_ESM.pdf]

Suppl Table 1

Supplementary Table 1. Kinase inhibitory activities for FRAX1036 tested at 1 μM concentration.

Inhibition	Kinases (n = 247, Invitrogen)
< 50%	Abl, Abl(E255K), ACVR1B, ACVR2B, AKT1, AKT2, ALK2, Arg, ASK1, Aurora_A, Aurora_B, Axl, Blk, BMPR1A, Bmx, B-Raf, B-Raf(V599E), BrSK1, BTK, CaMKI, CaMKI_delta, CamKII_alpha, CaMKII_beta, CamKIV, CAMKK1, CAMKK2, CDK1/cyclinB, CDK2/cyclinA, CDK5/p25, CDK5/p35, CDK7/cyclinH, CDK8/cyclinC, CDK9/cyclinT1, CHK1, CHK2, CK1_alpha1, CK1_delta, CK1_gamma1, CK1_gamma2, CK2_alpha1, CLK1, CLK2, CLK3, CLK4, Cot, CSF1R, CSK, DAPK1, DCAMKL2, DDR1, DMPK, DNA-PK, DRAK1, DYRK1A, DYRK1B, DYRK3, DYRK4, eEF-2K, EGFR, EGFR(L858R), EGFR(T790M), "EGFR(T790M, L858R)", EphA2, EphA3, EphA7, EphB3, EphB4, ErbB2, ErbB4, ERK1, ERK2, FAK, Fes, FGFR1, FGFR2, FGFR3, FGFR4, Fgr, Flt1, Flt3, Flt4, Frk, GRK2, GRK3, GRK5, GRK6, GSK3_alpha, GSK3_beta, HIPK1, HIPK2, HIPK4, Hyl, IGF1R, IKK_alpha, IKK_beta, IKK_epsilon, InsR, IRAK1, IRAK4, IRR, Itk, JAK1, JAK2, JAK3, JNK2, JNK3, KDR, Kit, LIMK1, LRRK2, LTK, Lyn, LynB, MAPKAPK2, MAPKAPK3, MARK1, MARK3, MEK1, MEK2, MEK3, MEKK2, MELK, Mer, Met, MKK6, MKNK1, MKNK2, MLK1, MLK2, MRCK_alpha, MSK1, MSSK1, MST1, MST2, mTOR, MuSK, MYLK(smMLCK), MYLK2(skMLCK), MYLK3(caMLCK), NEK1, NEK2, NEK4, NEK6, NEK9, p38_alpha(direct), p38_beta, p38_delta, p38_gamma, p70S6K, PAK4, PAK6, PAK7, PASK, PDGFR_alpha, PDGFR_alpha(V561D), PDGFR_beta, PDK1(direct), PhK_gamma1, PhK_gamma2, PI3K-A, PI3KC2b, PI3K-G, PIM1, PIM2, PKA, PKC_alpha, PKC_beta1, PKC_delta, PKC_epsilon, PKC_eta, PKC_theta, PKC_zeta, PKG1_alpha, PLK1, PLK2, PLK3, PRAK, PRK1, PRKAA1, PRKAA2, PrKX, "RAF1(Y340D, Y341D)", Ret, Ret(Y791F), ROCK1, ROCK2, Ron, Ros, Rse, Rsk1, Rsk2, Rsk3, Rsk4, SGK1, SGK2, SGK3, SLK, SPHK1, Src, Srm, SRPK1, STK16, STK33, Syk, TAK1-TAB1, TBK1, TEC, TGFBR1, TNK2, TrkA, TrkB, TSSK1, TTK, TXK, TYK2, WEE1, WNK2, Yes, ZAK, ZAP-70, ZIPK
50-75%	ARK5, EphA8, EphB1, GCK, JNK1_alpha1, MAP4K4, Mink1, NLK, RIPK2
> 75%	Brk, CK1_epsilon1, EphA1, KHS1, Lck, MST3, MST4, PAK1, PAK2, PAK3, PKD1, SIK2, TAO1, Tie2, YSK1
