# Supplementary material for: Small molecule inhibition of group I p21-activated kinases in breast cancer induces apoptosis and potentiates the activity of microtubule stabilizing agents
Source: Breast Cancer Res. 2015 Apr 23;17(1):59. doi: 10.1186/s13058-015-0564-5 (PMC4445529; doi:10.1186/s13058-015-0564-5)
Supplement: Additional file 3: Figure S2. — PAK1 levels and modulation of stathmin phosphorylation in breast cancer cells. (A) The protein expression level of PAK1, PAK2 and phospho-PAK1/2 were detected and compared in 15 breast cells. HCC2911 and MDA-MB-175 cells showed higher PAK1 activity and expression level. (B) FRAX1036 was administered to SUM52PE and T47D breast cancer cells at 0, 0.6, 1.2, 2.5, 5 and 10 μM for 24 hours. DMSO alone was used as a control. [file 13058_2015_564_MOESM3_ESM.pptx]

## Slide 1
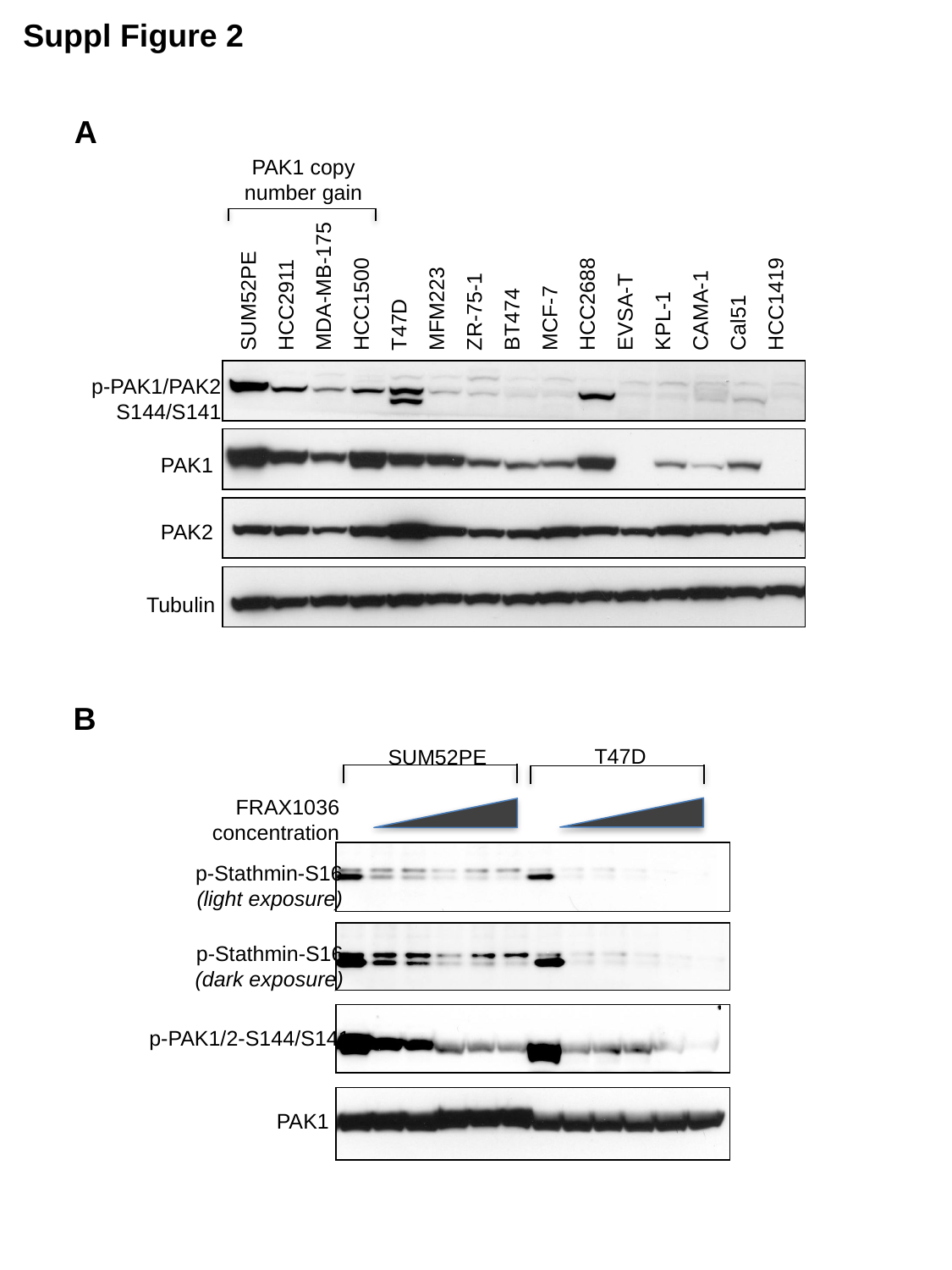

Suppl Figure 2
A
SUM52PE
HCC2911
MDA-MB-175
HCC1500
T47D
MFM223
ZR-75-1
BT474
MCF-7
HCC2688
EVSA-T
KPL-1
CAMA-1
Cal51
HCC1419
PAK1 copy number gain
p-PAK1/PAK2
S144/S141
PAK1
PAK2
Tubulin
B
T47D
SUM52PE
FRAX1036
concentration
p-Stathmin-S16
(light exposure)
p-Stathmin-S16
(dark exposure)
p-PAK1/2-S144/S141
PAK1
