# Supplementary material for: Small molecule inhibition of group I p21-activated kinases in breast cancer induces apoptosis and potentiates the activity of microtubule stabilizing agents
Source: Breast Cancer Res. 2015 Apr 23;17(1):59. doi: 10.1186/s13058-015-0564-5 (PMC4445529; doi:10.1186/s13058-015-0564-5)
Supplement: Additional file 4: Figure S3. — Combination effects of FRAX1036 and docetaxel (DTX) on cell viability assay in HCC2911 and MDA-MB175 cells. (A) HCC2911 and (B) MDA-MB175 cells were treated with FRAX1036, DTX, or the combination of both drugs and measured in a 4-day Cell Titer-Glo assay. Percentage of inhibition for cell viability and ΔBliss data were calculated and plotted as a dose range for both drugs. [file 13058_2015_564_MOESM4_ESM.pptx]

## Slide 1
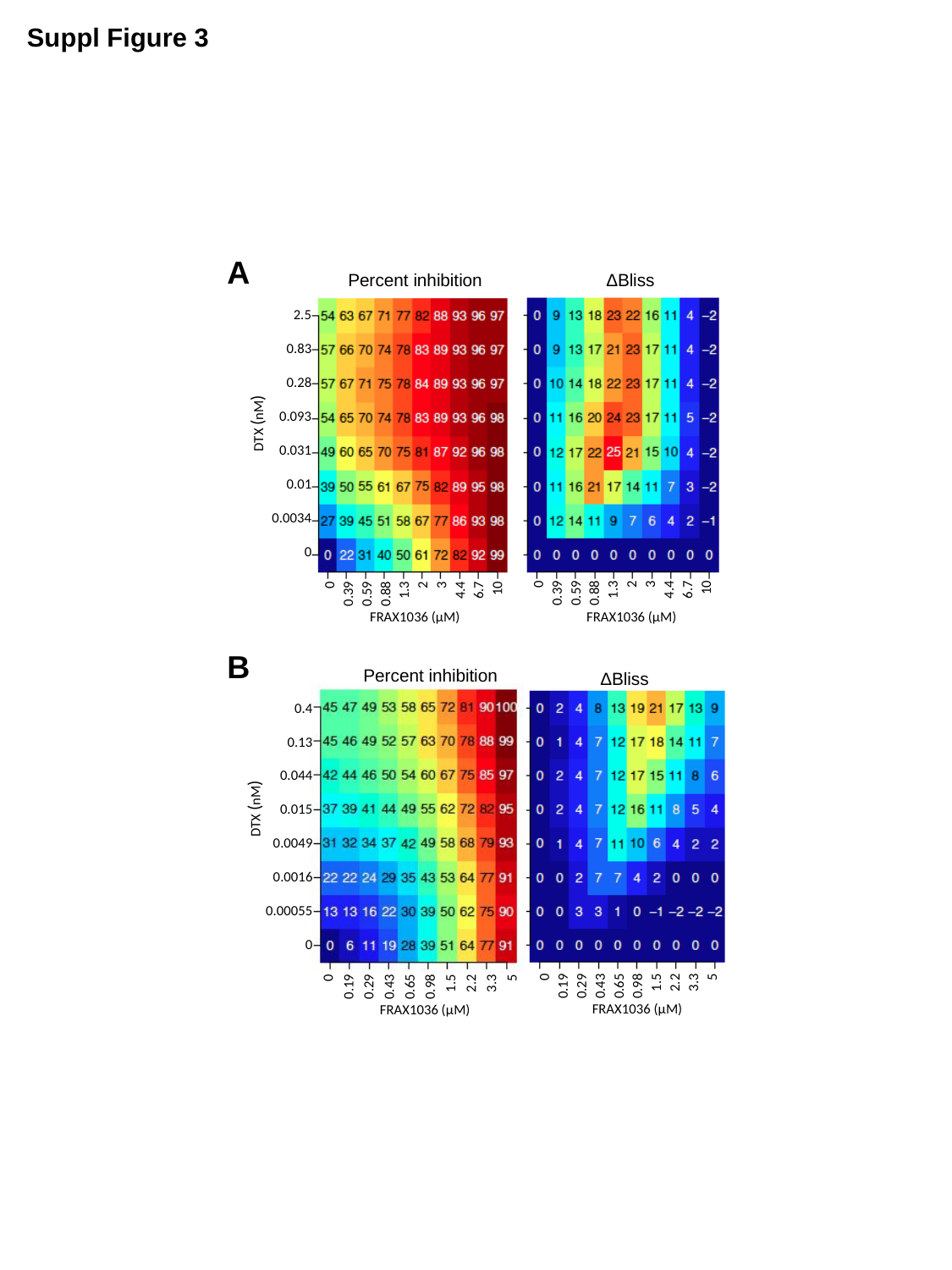

# Suppl Figure 3
A
Percent inhibition
ΔBliss
FRAX1036 (μM)
FRAX1036 (μM)
DTX (nM)
2.5
0.83
0.28
0.093
0.031
0.01
0.0034
0
0
0.39
0.59
0.88
1.3
2
3
4.4
6.7
10
0
0.39
0.59
0.88
1.3
2
3
4.4
6.7
10
B
Percent inhibition
ΔBliss
0.4
0.13
0.044
0.015
0.0049
0.0016
0.00055
0
DTX (nM)
0
0.19
0.29
0.43
0.65
0.98
1.5
2.2
3.3
5
0
0.19
0.29
0.43
0.65
0.98
1.5
2.2
3.3
5
FRAX1036 (μM)
FRAX1036 (μM)
