# Supplementary material for: Small molecule inhibition of group I p21-activated kinases in breast cancer induces apoptosis and potentiates the activity of microtubule stabilizing agents
Source: Breast Cancer Res. 2015 Apr 23;17(1):59. doi: 10.1186/s13058-015-0564-5 (PMC4445529; doi:10.1186/s13058-015-0564-5)
Supplement: Additional file 5: Figure S5 — Kinetic apoptosis assay and live-cell microscopy were used to further determine the extent of apoptosis in response to FRAX1036, docetaxel (DTX), and a combination. (A) Kinetic apoptosis assay of U2OS cells treated as in Figure 3 and imaged every 2 hours. Average and SEM of three replicates are shown (*P < 0.01). (B) U2OS cells were treated with 2 μM FRAX1036 and 0.2 μM DTX as indicated for 24 hours. Lysates were analyzed for proximal and distal pharmacodynamics biomarkers. (C) Time lapse images of U2OS cells stably expressing RFP-Tubulin and GFP-Histone H2B after treatment with DMSO, 2.5 μM FRAX1036, 0.2 μM docetaxel, or combination of 2.5 μM FRAX1036 and 0.2 μM DTX. Images are overlays of phase, RFP and GFP of a single field of view showing microtubules, nuclei and cell morphology from time-lapse movies (Additional file 7: Movie 1) at each time point. Symbols highlight different cell fates quantified in Figure 4C (*Mitotic; +slipped with micronuclei; ^apoptotic). Scale bar = 20 μm. [file 13058_2015_564_MOESM5_ESM.pptx]

## Slide 1
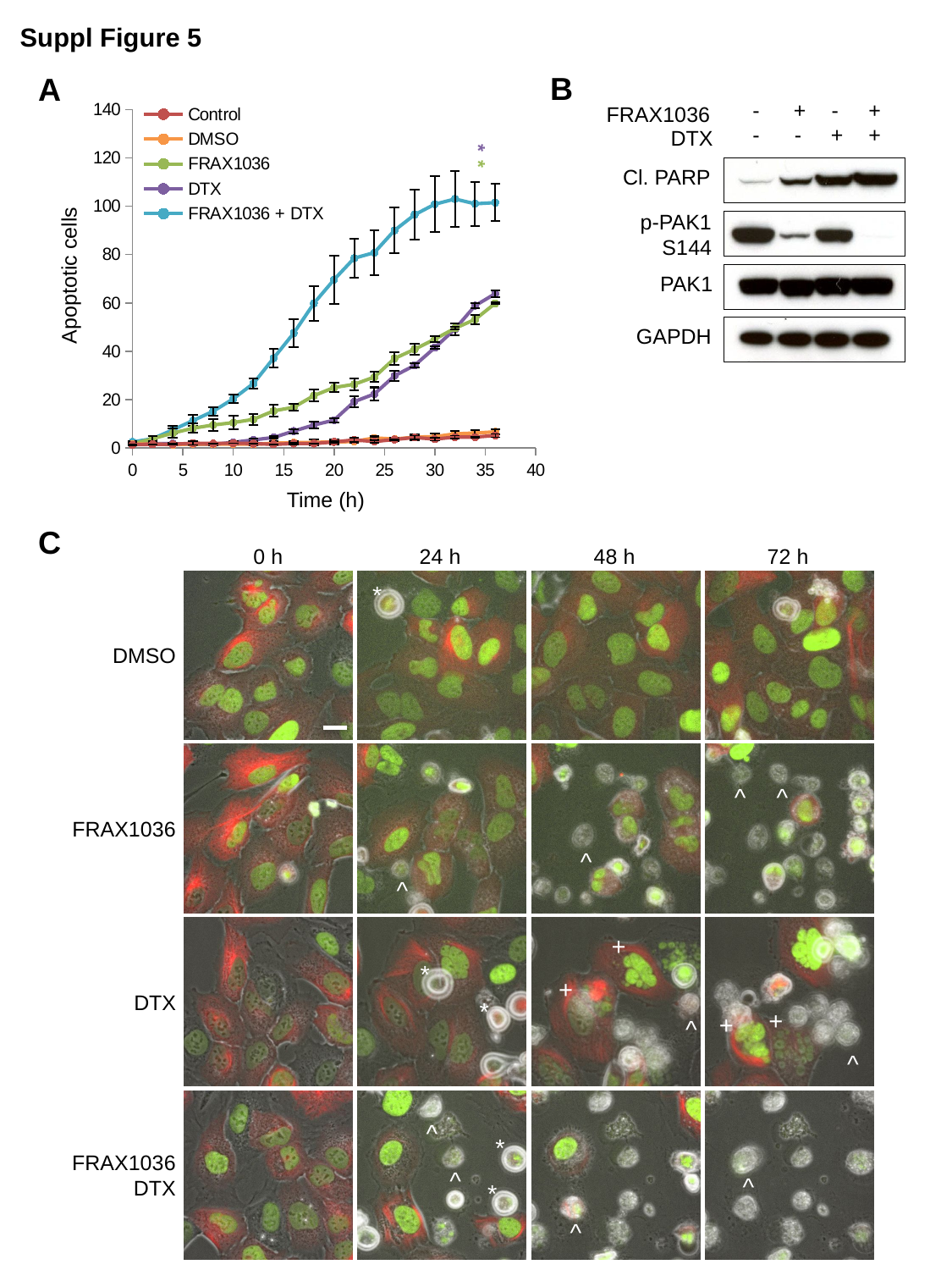

# Suppl Figure 5
B
A
-
+
-
+
FRAX1036
-
-
+
+
DTX
Cl. PARP
p-PAK1
S144
PAK1
GAPDH
### Chart
| Category | | | | | |
|---|---|---|---|---|---|Apoptotic cells
Time (h)
*
*
C
0 h
24 h
48 h
72 h
*
DMSO
FRAX1036
^
^
+
*
+
DTX
*
+
+
^
^
*
FRAX1036
DTX
^
^
*
^
^
^
^
