# Supplementary figures and images for: Small molecule inhibition of group I p21-activated kinases in breast cancer induces apoptosis and potentiates the activity of microtubule stabilizing agents
Source: Breast Cancer Res. 2015 Apr 23;17(1):59. doi: 10.1186/s13058-015-0564-5 (PMC4445529; doi:10.1186/s13058-015-0564-5)

## Slide 1
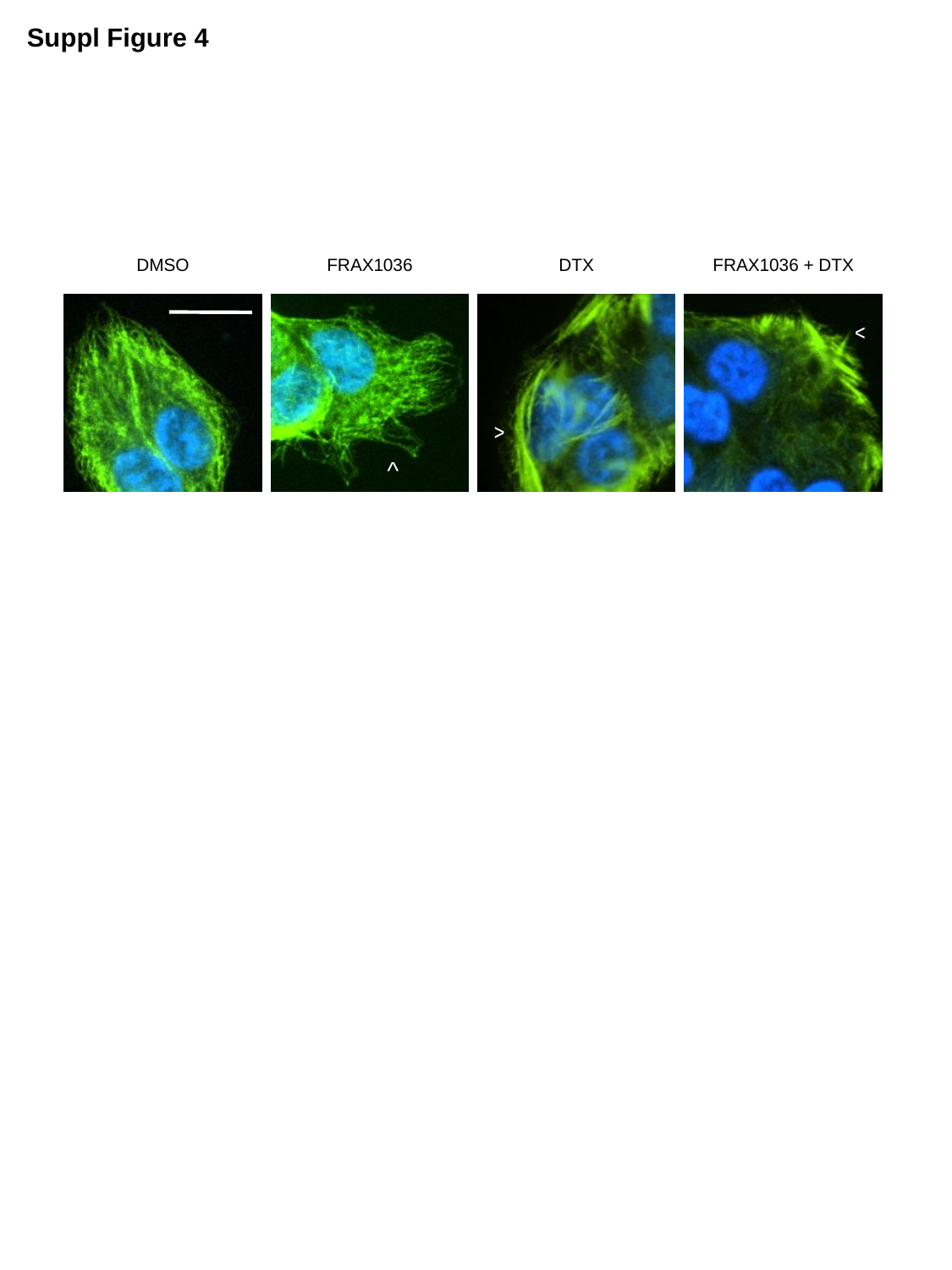

# Suppl Figure 4
DMSO
FRAX1036
DTX
FRAX1036 + DTX
^
^
^

Supplement: Additional file 6: Figure S4. — Microtubule organization in MDA-MB-175 cells treated with FRAX1036 and DTX. Spinning-disk confocal immunofluorescence images of fixed MDA-MB-175 cells. Cells were treated with DMSO, 2.5 μM FRAX1036, 0.2 μM DTX, or a combination of 2.5 μM FRAX1036 and 0.2 μM DTX for 24 hours before fixation. Scale bar = 20 μm. [file 13058_2015_564_MOESM6_ESM.pptx]
