# Supplementary material for: Small molecule inhibition of group I p21-activated kinases in breast cancer induces apoptosis and potentiates the activity of microtubule stabilizing agents
Source: Breast Cancer Res. 2015 Apr 23;17(1):59. doi: 10.1186/s13058-015-0564-5 (PMC4445529; doi:10.1186/s13058-015-0564-5)
Supplement: Additional file 9: Figure S6. — FRAX1036 and docetaxel order-of-addition modulates cell viability in vitro. Cell viability was quantified 48 hours following FRAX1036 and docetaxel (DTX) administered either simultaneously (bar 4), FRAX1036 preceding DTX by 4 hours (bar 5) or DTX preceding FRAX1036 by 4 hours (bar 6). The average and SEM of three replicates are shown. Simultaneous dosing and DTX followed by PAK1 inhibitor treatment were most efficacious (**P < 0.001). [file 13058_2015_564_MOESM9_ESM.pptx]

## Slide 1
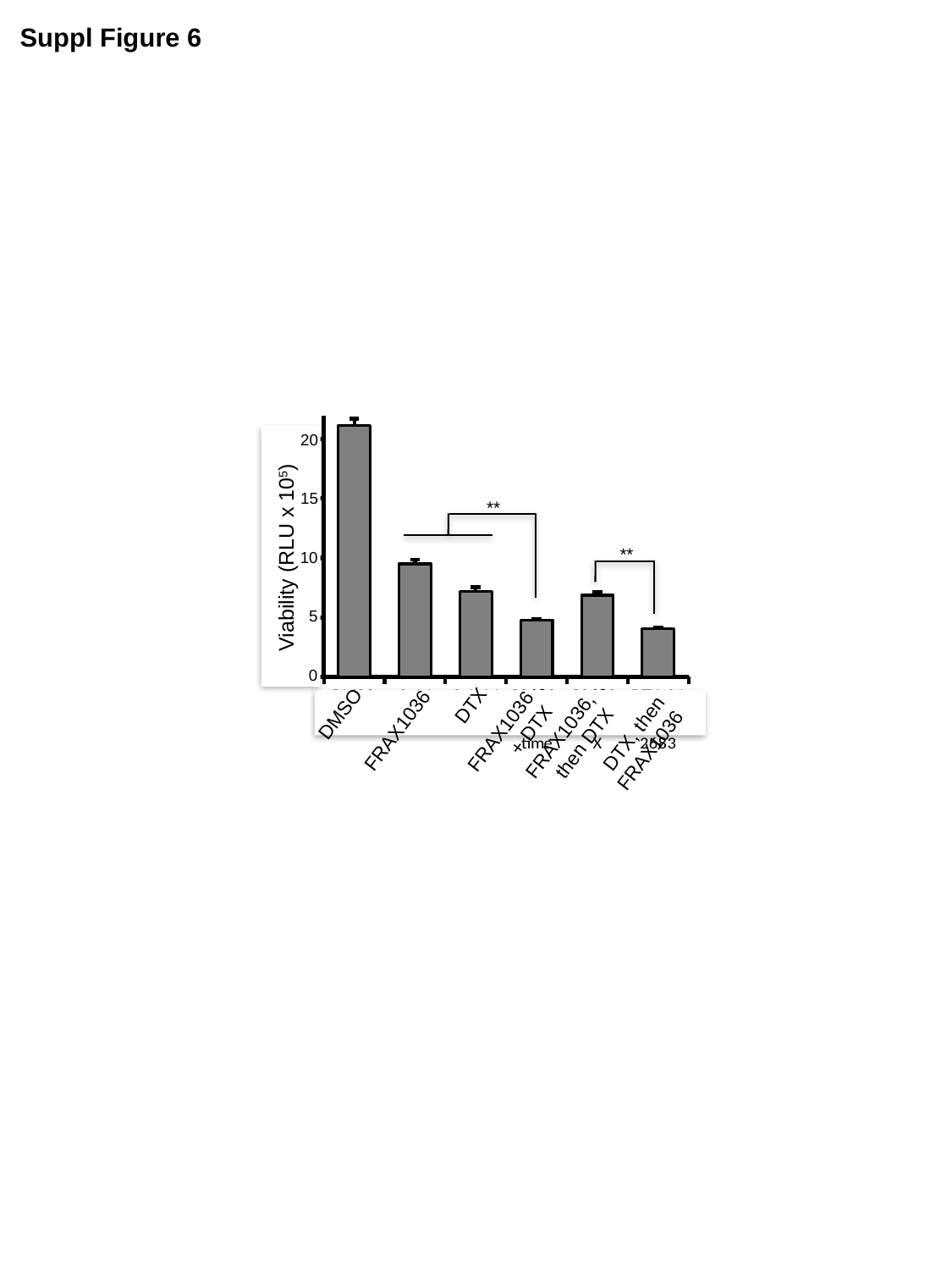

Suppl Figure 6
### Chart
| Category | |
|---|---|
| DMSO | 211474.0 |
| 2uM G2685 | 95084.0 |
| 0.1uM DTX | 71621.0 |
| G2683_DTX same time | 47653.0 |
| G2683_24hrs later_DTX | 68680.5 |
| DTX_24hrs later_G2683 | 40589.75 |20
15
Viability (RLU x 105)
10
5
0
DTX
DMSO
FRAX1036
+ DTX
FRAX1036,
then DTX
DTX, then
FRAX1036
FRAX1036
**
**
